# Target product profile for the development of pediatric formulations of new drugs for the treatment of children with *T. cruzi* infection

**DOI:** 10.1371/journal.pntd.0013597

**Published:** 2026-01-09

**Authors:** Colin Forsyth, Facundo Garcia-Bournissen, Guillermo Moscatelli, Samanta Moroni, Ana Pereiro, Lourdes Ortiz-Daza, Elvira-Idalia Hernández Cuevas, Freddy Tinajeros, Tayná Marques, Andrea Marchiol, Rafael Herazo, Sandra Seu, Javier Sancho, Jaime Altcheh, María-Jesús Pinazo

**Affiliations:** 1 Drugs for Neglected Diseases initiative, Rio de Janeiro, Brazil,; 2 Servicio de Parasitologia and Instituto Multidisciplinario de Investigacion en Patologias Pediatricas (IMIPP) CONICET, Hospital de Niños Ricardo Gutierrez, Buenos Aires, Argentina,; 3 Fundación Mundo Sano, Buenos Aires, Argentina; 4 Plataforma de atención integral a los pacientes con enfermedad de Chagas, Tariia, Bolivia; 5 Universidad Autónoma Juan Misael Saracho, Tarija, Bolivia; 6 Federación Internacional de Asociaciones de Personas Afectadas por la Enfermedad de Chagas (FINDECHAGAS), Mexico,; 7 Asociación Benéfica PRISMA, Lima, Peru; 8 Hospital Percy Boland Rodríguez, Ministerio de Salud Bolivia, Santa Cruz, Bolivia; 9 Dirección de Enfermedades Transmisibles por Vectores, Santiago del Estero, Argentina; 10 Chagas Disease Global Coalition, based in Barcelona, Spain; 11 Centro de Investigación Biomédica en Red de Enfermedades Infecciosas (CIBERINFEC), Instituto de Salud Carlos III, Madrid, Spain; Tulane University School of Public Health and Tropical Medicine, UNITED STATES OF AMERICA

## Abstract

**Background:**

Infection with the protozoan *Trypanosoma cruzi* leads to Chagas disease, a neglected tropical disease with potentially serious complications. Infection commonly takes place in childhood which can lead, without timely diagnosis and accurate treatment, to severe cardiac events and early mortality. For many years there were no pediatric formulations. Currently, there are two very effective treatments. Nevertheless, new drugs and shorter treatments are desirable. Therefore, the aim of this report is to provide a target product profile (TPP) to guide the development of pediatric formulations of new drugs for the treatment of children with Chagas disease.

**Methods:**

A review of the relevant literature, focusing on the therapeutic options available for pediatric Chagas disease, was undertaken prior to setting up a TPP working group of recognized leaders in the field. After several drafts, the TPP was established through a consensus agreement by the group. For each attribute, essential and ideal requirements were defined, which specified the required TPP performance and use characteristics.

**Results:**

The TPP for the development of formulations of new drugs for treatment of children with *T. cruzi* infection includes 15 different attributes. The target population was defined as newborns to adolescents up to 18 years. The essential indication for the target product would cover acute/congenital and chronic *T. cruzi* infection; the ideal indication would include chronic indeterminate, congenital and/or acute vector-borne *T. cruzi* infection; people who are immunosuppressed, and pregnant and breastfeeding women. An age-appropriate drug formulation, acceptable palatability, dose, and ease of administration were considered to be particularly important for children.

**Conclusions:**

The TPP provides guidance for essential and ideal characteristics of formulations of new drugs for the treatment of children with *T. cruzi* infection.

## Author summary

Chagas disease is a neglected tropical infection with serious health consequences. Patients often acquire the infection in childhood from a mother that is living with the infection, while still in the womb. Two drugs appeared during the 60s and 70s that proved to be very effective, particularly during childhood. Since that time, new drugs have been tested but none appeared to have the same efficacy. A sixty-day treatment, alongside different barriers to accessing early diagnosis, leads to very low treatment rates, in particular for children. This is a problem because treatment is most effective in the early stage of infection. New drugs need to be developed to prevent Chagas disease causing serious and life-threatening health issues later in life. Developing pharmaceuticals for children can be challenging, with many important issues to consider, including how the drug is administered, length of treatment time, and the taste. We describe a target product profile produced by a panel of experts, which provides guidance on the important attributes of formulations of potential new drugs for treating Chagas disease in infants and children.

## Introduction

Vectorial transmission of Chagas disease mainly occurs during childhood in endemic areas. However, transmission can also occur through blood transfusion, organ transplantation, consumption of contaminated food or beverages, and from mother to fetus [1,2]. Maternal health and the risk of infection to newborns are an important but often ignored aspect of neglected tropical diseases (NTDs) in general and Chagas disease in particular. Chagas disease is caused by the protozoan parasite *Trypanosoma cruzi*, endemic to the Americas, with the main vectors for transmission being triatomine bugs [3]. Mother-to-child transmission is the main route of new *T. cruzi* infection; around 1.7 million women of childbearing age worldwide are estimated to have *T. cruzi* infection, resulting in 9,000 babies born with Chagas disease every year [4]. Congenital infection can occur in non-endemic areas, which is a major challenge for Chagas disease control in areas with no vector transmission [5]. Fortunately, treatment of women before they conceive (e.g., in adolescence) has been demonstrated to prevent congenital Chagas disease transmission [6–10]. However, the risk of infection in children through both vectorial and congenital transmission remains high. While acute *T. cruzi* infection is usually mild or symptom-free, approximately 30% of chronic cases will develop serious complications many years later, that besides being associated with a variable morbidity, in some cases limiting the life of the person, potentially resulting in early mortality [11]. Treatment in the acute phase is required to clear *T. cruzi* infection [12].

There are currently only two drugs available to treat *T. cruzi* infection, benznidazole and nifurtimox [13]. However, both drugs were developed over 50 years ago and have limitations to their use, especially during the chronic stage of the infection [14–16], mainly due to the incidence of related adverse drug reactions (ADRs) (most of them mild, but in 20% of cases these prevent completion of treatment), and due to their variable efficacy depending on the stage of the disease. Although efforts have been made to produce and provide pediatric formulations, some barriers persist that may justify exploring new formulations [1,17]. Innovation for new and better drugs should be a priority in order to promote early treatment and universal access [13].

Target product profiles (TPPs) define the required product attributes of new drugs [18]. They can help as a planning tool to place patients, such as pediatric populations, at the center of drug discovery. TPPs are intended to guide all interested parties (pharmaceutical companies, academia, research institutions, product development partnerships, non-governmental and civil society organizations, and donors) to achieve better organized and more successful drug development programs [19], helping pinpoint the clinical and economic benefits of new drugs and formulations [20]. TPPs are available for Chagas disease and highlight areas where diagnostics or treatment are needed, such as point-of-care diagnosis and assessment of response to treatment [21], and to define biomarkers for early assessment of treatment efficacy [22]. There is also a TPP for Chagas disease treatment [23], however, given the particular needs of children, it was considered important to develop a specific TPP for the pediatric population infected with *T. cruzi* to help guide the development of appropriately adapted new medicines and therapeutic approaches. In this article, we present a TPP developed by an expert panel.

## Methods

The starting point for this TPP was a review of relevant literature on the treatment of children with *T. cruzi* infection, including the work published on pharmacology related to *T. cruzi* infection treatment. We conducted a narrative review of the literature; the search was conducted in PubMed and incorporated search terms including Chagas disease, target product profile, pediatric, *Trypanosoma cruzi*, pharmacokinetics, and treatment, without time limitations. We excluded sources not related to Chagas disease or not directly applicable to treatment or to target product profiles (e.g., studies of the vector).

Recognized leaders in the field were then invited to contribute to the TPP working group based on their proven expertise (see authors/acknowledgements for a full list). The panel was selected among experts in the Chagas Clinical Research Platform, which is a global network of over 400 experts and other stakeholders dedicated to Chagas disease research and treatment access. Researchers included on the panel were experts from a wide range of geographical regions and backgrounds, including representatives of people at risk of having *T. cruzi* infection, pediatricians with experience in managing patients with *T. cruzi* (caregivers), researchers from different backgrounds (e.g., clinicians and pharmacologists) experts in pediatric clinical studies, healthcare personnel with expertise in in access and implementation of care models, medical anthropologists and epidemiologists, and experts in communication and advocacy linked to the *T. cruzi* infection community. After the literature review, a first TPP draft was then prepared and based on the feedback in the first round, another draft was prepared and circulated among the participants of the working group. This was then repeated over several rounds, which allowed all contributors to provide critical input and suggest points for review. Additionally, the draft Target Product Profile was made available and feedback was invited via the Chagas Clinical Research Platform (both at its annual plenary meeting and via its web forum) before being finalized. A final draft was prepared after consideration of all feedback and discussion points in advance of a consensus meeting with experts and relevant stakeholders (representatives of, or people at risk of, suffering from *T. cruzi* infection/ Chagas disease). The final consensus TPP was then established after unanimous agreement by the group.

Given the lack of published data on the topic, the minimum and optimal requirements for each attribute were defined through consensus as “essential requirements”. These represent target characteristics of a candidate regimen that are the same as or better than the current standard-of-care and are considered an acceptable minimum for global health impact; they provide the basis for making critical decisions throughout the development and assessment process. In addition, the “ideal requirements” were defined for a product, which, if met, would broaden and accelerate the global health impact. Any future regimen should have at least all the essential requirements and as many of the ideal requirements as possible.

## Results

The TPP for the treatment of children with *T. cruzi* infection is summarized in Table 1 and includes 15 different attributes. The working group identified both essential and ideal indications for the development of pediatric formulations. The essential indication is for children with acute/congenital and chronic *T. cruzi* infection; however, the ideal indication would include children with chronic indeterminate, congenital, and/or acute vector-borne *T. cruzi* infection, immunosuppressed people, and pregnant women. The target population was defined as newborns to adolescents (age 0–18 years).

**Table 1:** TPP for the development of pediatric formulations of new drugs for the treatment of Chagas disease.

	Essential	Ideal
**Indication**	Children with acute/congenital and chronic vector-borne *T. cruzi* infection.	Children with chronic, congenital and/or acute vector-borne *T. cruzi* infection; immunosuppressed. Pregnant women.^a^
**Population**	Newborns to adolescents	Newborns to adolescents
**Safety**	Tolerance equal to or better than standard treatments. Absence of long-term toxicity (e.g., absence of toxicity to bone metabolism, neurodevelopment, or reproductive toxicity).	Better tolerance than standard treatments. Safety in pregnancy (i.e., no genotoxicity and teratogenic risk, no increased risk of miscarriage or preterm birth, or other pregnancy complications) and breastfeeding.
**Pre-clinical efficacy**	*In vitro* activity and clinical evaluation in *T. cruzi* (DTU^b^ 1 and DTU^b^2)	*In vitro* activity and clinical evaluation against all DTUs^b^ in different geographical areas.
**Efficacy**	Therapeutic response greater than 75% (serological negativization/seroreduction of at least 20% at 12 months) and persistent negative results of parasitological^c^ test(direct and/or molecular parasitological methods).	Therapeutic response greater than 90% (serological negativization, or sero reduction of at least 20% at 12 months) and persistent negativization of parasitemia (direct and/or molecular parasitological methods).
**Formulation**	Slotted dispersible tablet liquid formulation injectable formulation (for critical cases). Simple dosing, no need for formulation manipulation.	
**Palatability**	Pleasant taste or taste masked	Pleasant taste or taste masked
**Dosing Frequency**	Twice a day	Once perday or less
**Dose flexibility**	Able to easily measure and administer the required dose (adjusted by weight when needed) to all patients	Able to easily measure and administer the required dose (adjusted by weight when needed) to all patients
**Duration**	8-30 days	7 days
**Administration**	Oral & Injectable IV No significant interaction with food (unmodified absorption)	
**Stability & storage**	Tolerance for light, humidity, and tropical temperatures with proper packaging, no refrigeration required. Stable for at least 2 years.	Tolerance for light, humidity, and tropical temperatures even without packaging, no refrigeration required. Stable for at least 5 years.
**Packaging**	Childproof, label visually informative and easy to understand, or for people who do not read. Blister pack, syrup	Childproof, label visually informative and easy to understand, or for people who do not read. Blister pack, syrup.
**Drug interactions**	No major interactions with medications commonly used in pediatrics and oral contraceptives.	No serious interactions with other medications, including oral contraceptives.
**Access & cost**	Cost appropriate to the cost of production. Widely available in the health system, and out-of-hospital pharmacies. Guaranteed distribution and production in the long term.	Cost appropriate to the cost of production. Widely available in the health system, manageable in primary care centers and other health system centers, and out-of-hospital pharmacies. Guaranteed distribution and production in the long term.
**Registration**	All Latin American regulatory agencies, and international regulatory agencies (FDA^d^-EMA^e^).	All Latin American regulatory agencies, and international regulatory agencies (FDA-EMA), and in the main non-endemic countries with cases of Chagas.

Notes: ^a^ Women in this context refers to post-pubertal girls; ^b^ DTU refers to Discrete Type Unit of *Trypanosoma cruzi* reflecting phylogenetic lineages; ^c^ persistent negative results of parasitological is defined as negative PCR results at the end of treatment and at least one year postreatment evaluation; ^d^ Food and Drug Administration of the United States of America; ^e^ European Medicines Agency.

## Discussion

This TPP was developed to present the essential and ideal characteristics of pediatric formulations of new drugs for the treatment of Chagas disease. The expert working group identified 15 attributes that need to be considered by anyone developing Chagas disease formulations for children, including the target population, safety, efficacy, formulation, palatability, and administration.

Even if in 2017 DNDi and collaborators developed a TPP for Chagas disease treatment with a more general approach, and even if key attributes overlap partially with the ones described at that time, this TPP adds more details to the definition of the essential and ideal attributes for the development of medicines for paediatric use. Besides, the paediatric TPP prioritizes congenital and acute infection, and also includes the cases of pregnant adolescents and women. It also stresses the relevance of including aspects linked to access (taking also into consideration attributes like stability and storage) and costs, as well as global regulatory expectations for a new medicine under development to be registered and adopted.

Without accurate treatment, children are at risk of developing Chagas disease, which, later in life, may cause serious disease and early mortality. There is strong evidence to support the recommendation to treat infants with *T. cruzi* infection within the first year of life [24,25]. Given the efficacy of the two drugs that are available, there are no medical reasons to avoid treating children with Chagas disease. However, new drugs for Chagas disease therapy should be developed in order to eliminate any barriers related to their use [13,14,26]. If new drugs for Chagas disease are going to be suitable for children, their formulations need to be carefully considered.

Current treatments with benznidazole and nifurtimox are generally well tolerated, with up to 90% cure rates in infants [27–30]. Unfortunately, there is limited data on the safety of the current treatments in terms of their genotoxicity and teratogenic risk, and for this reason they are commonly avoided in pregnant women. Thus, screening to identify women with the infection, ideally before pregnancy, and diagnosis, monitoring, and treatment of infants with the infection is strongly recommended [31]. Factors limiting the use of adult-focused formulations (i.e., tablets) in children have been overcome through clinical programs that were set up to develop pediatric formulations of benznidazole and nifurtimox [32,33]. However, in some countries, the pediatric formulations of these drugs have not been registered by the pharmaceutical companies who developed them, and children must receive treatment off-label. Moreover, currently less than 1% of people with *T. cruzi* infection receive a treatment regimen regardless of their age [17,26,34,35]. It is important to note that the process of registering a drug is very arduous, as is maintaining it: if there is only a small number of patients in each country ready to receive such a treatment, industry will be discouraged from engaging, making alternative solutions, such as PAHO-mediated donation, a more realistic approach today. The reasons for the low treatment rate are complex, but include ambiguous messaging about treatment benefits, scarce knowledge of the disease, and limited and inconsistent access to drugs for different and context specific reasons [17,35]. Patients may have difficulty getting medication even in areas where the drugs have been approved, such as in the USA [1,36]. In endemic areas, particularly poor rural regions, access to healthcare providers can be difficult, and the availability of medications is low and unpredictable, which is particularly pertinent because Chagas disease treatment requires 30–60 days of dosing. Barriers into access related to economics have been mitigated by donation to countries and free delivery by health authorities that buy the drug. Without these measures, costs could be a barrier for people with low income [37]. The gap between the demand for etiologic treatment and the estimates of disease prevalence in endemic areas of Latin America demonstrates that access to treatment is lacking [35]. Up to 2012 there were disruptions in the supply of benznidazole and nifurtimox due to insufficient levels of production and disruption in the supply chain [17]. From 2012 to the present, production has been assured. Children have further hurdles to overcome, such as misinformation about the availability, use, and safety of pediatric formulations.

Alongside access to the drugs, awareness of the disease among patients and healthcare providers remains below optimal, despite efforts at publicizing Chagas disease, meaning that many people are unaware of their risk of infection. Early detection and treatment of infection in children are vital to prevent disease development. Difficulties with the treatment regimen are also a factor. Adverse drug reactions, absence of treatment algorithms to address these, and long courses of therapy are usually mentioned as barriers for treatment. Since other drugs with more ADRs are commonly used, it is clear there is a complex network of different barriers surrounding the treatment of Chagas disease [37]. The need for novel drugs, repurposed drugs, or combination therapies that are safer and more effective in chronic infection is evident [26], but in the meantime the current therapies are still needed.

Discovery of new drugs is often challenging, with high attrition rates even in areas with high pharmaceutical investment and where a significant return on investment is expected. In an environment where the number of clinical trials involving children is already limited, drug development for pediatric populations with NTDs can be particularly challenging [38]. However, this approach is changing, and pediatric drug development is becoming increasingly important, and in some jurisdictions, mandatory or strongly encouraged. Several regulatory incentives and resources are now aimed at addressing these needs and promoting the development of formulations for children [39].

To guide research efforts towards the development of new therapeutic options to treat *T. cruzi* infection in children, we present a TPP for Chagas disease that aims to develop an oral, age-adapted shorter-course treatment that is safer and more effective than current options, for use in all regions and in both chronic and acute patients, including during pregnancy [23]. However, it is important to acknowledge that the pediatric population has some distinct needs that merit a standalone TPP, and guidance for this goal has been published [40]. Several addition to the previous TPP, built for a more general population, were highlighted above, but it is important to note that additional pharmaceutical specificities, such as more detailed formulation criteria for medicines to be used for children were added to this new TPP. Different patient populations have different pharmacological profiles, such as the lower plasma concentration of benznidazole in children [27]. While the tendency to exclude young children, adolescents, and pregnant and lactating women from clinical trials is changing, the numbers of these patients in recent studies are still low, so there is an inevitable delay in the approval process [41]. Pediatric populations may be considered a neglected population, so it is important to prioritize the affordability, availability, and adaptability of drugs to suit them [41].

Many points in this TPP for children are similar to those suggested in a previous TPP [23], reflecting similarities between the needs of children and more general populations. However, the current pediatric TPP highlights the treatment of acute/congenital infection as essential, while the previous TPP was more focused on chronic aspects of the infection. This distinction is important because of the large number of neonates who contract *T. cruzi* infection through mother-to-child transmission every year [4].

Important at this point to highlight another of the differences that the paediatric TPP added to the TPP developed in 2017: the new TPP introduces defined efficacy thresholds, in contrast to the previous relative-to-standard approach. It is essential that the efficacy of any new drug is greater than 75% (similar to current treatments), while efficacy greater than 90% would be ideal. Due to low and intermittent parasitemia, negative serological results are still the gold standard of cure [42]. However, achieving negative serological results (defined as the gold standard of cure) in adults and in adolescents in the chronic stage of the infection is quite difficult because of the prolonged persistence (potentially for decades) of antibodies to *T. cruzi* and limitations in the sensitivity of parasitological tests [6]. Molecular techniques generally involving polymerase chain reaction (PCR) can now identify low levels of parasitemia, but their use in the chronic phase of *T. cruzi* infection remains debatable because of variability in results, and because they have been a marker of therapeutic failure rather than a test for cure. These test results depend on the degree of parasitemia, sample volume, DNA purification method, target region, patient characteristics, and parasite genetic variability [43]. In children the scenario is different. For congenital transmission, the diagnosis is based on direct observation or PCR test during the first month of the newborn’s life [44]. If parasitemia is negative, due to low parasite level, diagnosis is based on a serological test after maternal *T. cruzi* antibodies have disappeared, after 9–12 months of age [43]. There is also evidence of decreased or serological negativity shortly after treatment in children, so serology is a useful marker of response to treatment in this group [13,45,46].

Another added attribute included in the paediatric TPP is the *in vitro* activity and clinical evaluation characteristics. Response to evaluation by clinical trials in at least *T. cruzi*-discrete type unit 1 (DTU1) and DTU2 was determined as an essential characteristic. This reflects the infection risk in children beyond endemic areas through infection routes other than vector transmission, such as congenital infection. An ideal characteristic would be to evaluate the new drug against various DTUs in different geographical areas, thus widely assessing its usefulness.

In terms of safety, the essential characteristics identified were tolerance equal or better than standard treatments, and absence of long-term toxicity to bone metabolism, neurodevelopment, or reproductive toxicity, reflecting concerns about the long-term effects of medications on developing children, while the ideal characteristics were better tolerance than standard treatments, severe immunological non-toxicity, safety in pregnancy, and absence of chronic toxicity. The current treatments have some ADRs, including rash and pruritus, headache, myalgia, and gastrointestinal discomfort for benznidazole and anorexia and weight loss, irritability, sleepiness, and other nervous system signs and symptoms for nifurtimox [13,47]. These symptoms prevent patients from finishing the prescribed treatment regimen in around 20% of cases in adults and in 5% in children if they are not properly monitored by the health teams [13,47]. This is worth highlighting from the perspective of safety during pregnancy, because it will have an impact upon both the mother and newborn infant. The evidence shows that current treatments are safe for the baby during breastfeeding [48,49], however, extended information on the reproductive toxicity of benznidazole and nifurtimox is lacking. Given that effective treatment will prevent future congenital transmission, and that in some areas (e.g., rural impoverished areas) a young woman may only come in contact with the health system during pregnancy and delivery, providing treatment as soon as possible after delivery has the potential to prevent future infection of newborns, as would developing treatments that could be administered during pregnancy.

Drug-drug interactions are another safety-related aspect that must be characterized to ensure the product has no serious interactions with other medications, and in this TPP, in contrast to the TPP for general population developed in 2017, the definition of the essential and ideal characteristics is more accurate.

If the final goal is to expand the use of the new therapeutic options, other equally important characteristics must be taken into consideration. The formulation, palatability, and method of administration are particularly important to pediatric populations and impact the acceptability of treatment. Oral dosing is preferable for most children, and dosing needs to be simple with minimal or no need to break tablets [38]. Therefore, a liquid formulation may be considered, and this could be injectable for patients who cannot tolerate oral medications, such as premature newborns and immunosuppressed patients. However, the use of liquid may be limited in some locations due to storage and transportation issues. Liquid formulations are often less stable than tablets, weigh more, and can be difficult to transport, often requiring refrigeration in hot climates [50]. Dispersible tablets offer many benefits in terms of storage and can be easily swallowed in a liquid, while a grooved tablet allows for split dosing. An ideal formulation would be a slotted dispersible tablet with no need for formulation manipulation. In addition, there is a requirement for a pleasant taste to ensure the solution is palatable to infants and children [39].

Another important attribute adapted in this TPP for paediatric use (and another difference regarding the TPP for medicines of general use in Chagas) is the consensus to markedly shorten the ideal treatment duration (7 days) compared to the broader <30-day goal in the general one. Dosing frequency, included in this TPP and not in the previous one, was defined to be twice or ideally once daily, for a period of 8–30 days, and ideally 7 days, since long treatment durations can lead to discontinuation, which impacts efficacy and drug resistance [51]. Dose flexibility, with the ability to be adjusted and the requirement that the medicine should be easily measured is also requested as an attribute to be considered in drug development for children in Chagas context. Administration needs to be oral or injectable intravenously (IV), with no food interaction, as modified absorption can complicate dosing. Other attributes to take into consideration that were lacking in previous TPPs were: a) Stability and storage are also vital considerations in many low and middle-income countries (LMICs),and b) Packaging, which must also be childproof and provide information in a visual manner easy to understand, since those administering the treatment may not be able to read. A blister pack for the tablets, for example, would make them easy to store and administer.

Access and cost factors are particularly important considerations for a product targeting an NTD in a neglected population. In this case, it is essential that the cost of goods is aligned with the cost of production. The product should be widely available in the health system and out-of-hospital pharmacies. It would be ideal if the product were manageable in primary care centers and other health system centers. The current problems with access to treatment should not be repeated with new formulations, distribution and production must be guaranteed in the long term, and the use of future pediatric formulations promoted. Regarding regulatory expectations, an important difference with the previous TPP is the inclusion of a global regulatory ambition, setting the requirement that registration must be comprehensive to avoid the need for off-label use, including by Latin American and international agencies, such as the Food and Drug Administration of the United States of America (FDA) and the European Medicines Agency (EMA).

## Conclusion

We present a new TPP for the development of pediatric formulations of new drugs for *T. cruzi* infection. Besides efficacy and safety requirements, the TPP highlights specific characteristics that are relevant for children, such as drug formulation, palatability, dose, administration, and accessibility. This tool is intended to assist in the development of novel therapeutic options to fight Chagas disease in this especially vulnerable population, in which timely treatment can cure the infection, preventing the development of future organ damage.
